# Network Pharmacology and Experimental Validation Reveal the Potential Antidepressant‐Like Effects of Chaihu Shugan San Associated With Glutamate Homeostasis

**DOI:** 10.1002/fsn3.72384

**Published:** 2026-09-20

**Authors:** Yu Xu, Lian Xia, Keyun Zhou, Chuanxin Liu, Xiaoting Tian, Zongchun Yang

**Affiliations:** ^1^ School of Traditional Chinese Medicine Chongqing University of Chinese Medicine Chongqing China; ^2^ College of Traditional Chinese Medicine Chongqing Medical University Chongqing China; ^3^ School of Traditional Chinese Medicine Chongqing Medical and Pharmaceutical College Chongqing China

**Keywords:** Chaihu Shugan San, depression, GLT‐1, glutamate, network pharmacology, RAGE/NF‐κB pathway

## Abstract

Chaihu Shugan San (CHSGS) is a traditional Chinese formula widely used in the treatment of depression, but its antidepressant‐like effects remain incompletely understood. This study investigated the effects of CHSGS on stress‐induced behavioral abnormalities with a focus on hippocampal glutamate homeostasis. Ultra‐performance liquid chromatography–tandem mass spectrometry (UPLC‐MS/MS) was used to identify the chemical constituents of CHSGS, and network pharmacology combined with molecular docking was applied to predict potential targets and signaling pathways. A chronic restraint stress (CRS)‐induced mouse model was established to evaluate the antidepressant‐like effects of CHSGS through behavioral tests and molecular analyses. UPLC‐MS/MS identified flavonoids as the major constituents of CHSGS. Network pharmacology analysis suggested that the AGE–RAGE signaling pathway may be involved in the effects of CHSGS, with several flavonoids, including kaempferol, hesperetin, isorhamnetin, naringenin, and nobiletin, showing potential interactions with key targets. In CRS mice, CHSGS treatment improved behavioral abnormalities, reduced hippocampal glutamate levels, alleviated neuronal Nissl body damage, restored hippocampal GFAP and GLT‐1 expression, and inhibited RAGE/NF‐κB signaling activation. These findings suggest that CHSGS effectively alleviated CRS‐induced behavioral abnormalities and improved hippocampal glutamate homeostasis in mice, which may be associated with the regulation of GLT‐1 expression and involvement of the RAGE/NF‐κB signaling pathway.

## Graphical abstract

UPLC‐MS/MS and computational analyses identified the chemical constituents and potential biological relevance of CHSGS. In CRS‐exposed mice, CHSGS treatment alleviated behavioral abnormalities and improved hippocampal glutamate homeostasis, suggesting that regulation of glutamate homeostasis may contribute to its antidepressant‐like effects.

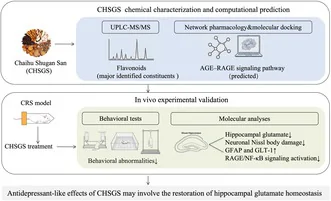

## 1 Introduction

Depression is a highly prevalent and recurrent psychiatric illness characterized by persistent low mood, anhedonia, sustained anxiety, cognitive impairment, and impaired social functioning. In severe cases, it may be accompanied by suicidal ideation and represents a major contributor to the global burden of disease (Abdoli et al. 2022). Current first‐line pharmacotherapies, including selective serotonin reuptake inhibitors (SSRIs) and serotonin–norepinephrine reuptake inhibitors (SNRIs), exert therapeutic effects primarily by increasing the availability of serotonin and norepinephrine. Although widely used in clinical practice, these agents are limited by delayed onset of action, often requiring days to weeks, as well as a considerable risk of relapse and recurrence (Harmer et al. 2017; Kato et al. 2021). Therefore, further exploration of alternative therapeutic strategies remains necessary.

Chaihu Shugan San (CHSGS), a classical traditional Chinese medicine (TCM) formula, consists of *Bupleuri Radix* (Chaihu), *Paeoniae Radix Alba* (Shaoyao), *Cyperi Rhizoma* (Xiangfu), *Aurantii Fructus* (Zhiqiao), *Citri Reticulatae Pericarpium* (Chenpi), *Chuanxiong Rhizoma* (Chuanxiong), and *Glycyrrhizae Radix et Rhizoma* (Gancao). It has been widely prescribed for the treatment of mood disorders. Recent clinical meta‐analyses have indicated that CHSGS is effective and generally safe for improving depressive symptoms (Zhang et al. 2024). Previous studies suggest that its antidepressant‐like mechanisms may involve modulating the monoaminergic neurotransmission, neuroinflammation, hypothalamic–pituitary–adrenal (HPA) axis dysfunction, and neurotrophic signaling (Guo et al. 2024; Wu et al. 2025). However, its antidepressant‐like effects remain insufficiently understood.

Disruption of glutamate homeostasis has increasingly been implicated in stress‐related depressive phenotypes. Astrocytes play a critical role in maintaining extracellular glutamate balance, primarily through excitatory amino acid transporter 2 (EAAT2), also known as GLT‐1, which mediates the majority of synaptic glutamate uptake (Rothstein et al. 1994). Chronic stress has been shown to downregulate GLT‐1 expression, potentially leading to excessive glutamate accumulation and subsequent neuronal injury (McIntyre and Jain 2024; Park and Choi 2025; Zhang et al. 2026). Several bioactive components of CHSGS have been reported to modulate GLT‐1 expression and alleviate neuronal injury (Li et al. 2020). Therefore, regulation of GLT‐1‐mediated glutamate transport may represent a potential mechanism contributing to the effects of CHSGS on glutamate homeostasis.

Network pharmacology combined with experimental validation has become a widely used strategy for elucidating the complex pharmacological networks of TCM formulations (Wang et al. 2021). Our preliminary network pharmacology analysis identified the AGE–RAGE signaling pathway as a candidate pathway associated with CHSGS (Li 2021). RAGE/NF‐κB signaling is involved in stress‐related neuroinflammatory responses and has also been associated with changes in GLT‐1 expression (Yang et al. 2020; Liu et al. 2022). However, its relationship with GLT‐1 regulation and glutamate homeostasis during CHSGS treatment remains uncertain. Therefore, the present study integrated ultra‐performance liquid chromatography–tandem mass spectrometry (UPLC–MS/MS) profiling, network pharmacology, molecular docking, and in vivo experiments to evaluate the antidepressant‐like effects of CHSGS in a chronic restraint stress (CRS) mouse model, with particular emphasis on glutamate homeostasis. This study aimed to provide new experimental evidence for the antidepressant‐like effects of CHSGS.

## 2 Materials and Methods

### 2.1 Preparation of CHSGS Solution and UPLC‐MS/MS Analysis

CHSGS, consisting of Chaihu (6 g), Chenpi (6 g), Chuanxiong (4.5 g), Xiangfu (4.5 g), Zhiqiao (4.5 g), Shaoyao (4.5 g), and Gancao (1.5 g), was provided and quality‐certified by Beijing Tongrentang Technology Development Co. (Beijing, China). The chemical constituents of CHSGS were identified and characterized using UPLC–MS/MS analysis. The CHSGS solution was transferred to a 1.5 mL centrifuge tube and extracted with 1 mL of 70% aqueous methanol, vortex‐mixed, and sonicated in a water bath for 90 min. After centrifugation at 16,000 × g for 10 min at 4°C, the supernatant was collected, freeze‐dried, reconstituted in 40% aqueous methanol, and centrifuged again at 16,000 × g for 10 min at 4°C. The resulting supernatant was subjected to UPLC–MS/MS analysis.

Chromatographic separation was achieved using a Vanquish UPLC system (Thermo Fisher Scientific, Germany) equipped with an ACQUITY UPLC HSS T3 column (2.1 × 100 mm, 1.8 μm; Waters, USA). The mobile phase consisted of water containing 0.1% formic acid (A) and acetonitrile (B) (LC–MS grade). Gradient elution was performed as follows: 5%–25% B (0–3 min), 25%–45% B (3–8.5 min), 45%–95% B (8.5–14 min), 95%–98% B (14–17 min), followed by a rapid decrease to 5% B (17–17.2 min) and equilibration at 5% B until 20 min.

Mass spectrometric analysis was performed using a Q‐Exactive HFX mass spectrometer (Thermo Fisher Scientific, Germany) with electrospray ionization (ESI) in both positive and negative ion modes. The spray voltages were set at 3800 and 3500 V, respectively. Data were acquired in Full MS/dd‐MS2 mode with resolutions of 60,000 for MS1 and 15,000 for MS/MS scans. Stepped normalized collision energies (NCE) of 20, 40, and 60 were applied, and the MS scan range was set to m/z 90–1300.

### 2.2 Network Pharmacology Analysis

The potential active compounds of CHSGS and their corresponding protein targets were retrieved from the Traditional Chinese Medicine Systems Pharmacology (TCMSP) database (Ru et al. 2014). Candidate compounds were screened based on oral bioavailability (OB) ≥ 30% and drug‐likeness (DL) ≥ 0.18. The corresponding protein targets were subsequently standardized to official gene symbols using the UniProt database, with the organism restricted to *Homo sapiens* , resulting in 908 CHSGS‐associated targets.

Depression‐related targets were collected from the Online Mendelian Inheritance in Man (OMIM) and GeneCards databases using “depression” as the search keyword. After removing duplicate targets, 1986 depression‐associated targets were retained. The overlapping targets between CHSGS‐related targets and depression‐associated targets were then identified using the EVenn online tool and considered candidate targets potentially associated with the antidepressant‐like effects of CHSGS.

A compound–target–disease network was constructed using Cytoscape software (version 3.9.1). Network topology parameters, including degree centrality, betweenness centrality, and closeness centrality, were calculated to evaluate the topological importance of network nodes. Gene Ontology (GO) functional enrichment analysis and Kyoto Encyclopedia of Genes and Genomes (KEGG) pathway enrichment analysis were performed using the DAVID database, and significantly enriched biological processes and signaling pathways were visualized.

### 2.3 Molecular Docking

Molecular docking was performed as described previously (Ren et al. 2024). Three‐dimensional structures of small‐molecule ligands were obtained from the PubChem database (http://pubchem.ncbi.nlm.nih.gov/), and the crystal structures of target proteins were retrieved from the RCSB Protein Data Bank (PDB; https://www.rcsb.org/). Molecular docking simulations were performed using AutoDock Vina. The predicted binding affinities of ligand–protein interactions were evaluated based on the docking scores (kcal/mol), and the optimal docking conformations were visualized using PyMOL.

### 2.4 Animals and Treatment Protocols

Healthy male BALB/c mice (8 weeks old, 20–25 g) were provided by Laboratory Animal Center of Chong Qing Medical University. All mice were housed in an environment maintained at 22°C–24°C, 40%–70% humidity, with a 12 h light/dark cycle. Animals had free access to food and water. The animal study protocol was approved by the Animal Ethics Committee of Chong Qing Medical University (approval number: IACUC‐CQMU‐2025‐0313).

After 5 days of adaptive feeding, mice were randomly divided into five groups (*n* = 6 per group): control, model, CHSGS‐L, CHSGS‐H, and fluoxetine groups. Chronic restraint stress (CRS) was induced as previously described in our study with minor modifications (Qian et al. 2020). Briefly, mice, except those in the control group, were individually restrained in 50 mL centrifuge tubes for 6 h per day (9:00 a.m. to 3:00 p.m.) for 21 consecutive days to induce stress‐induced behavioral abnormalities. During the CRS exposure period, mice in the CHSGS‐L and CHSGS‐H groups received daily intragastric administration of CHSGS decoction at doses of 6.5 and 13 g/kg body weight. These doses were selected based on our previous study of CHSGS and converted from clinically equivalent doses (Qian et al. 2020). Mice in the fluoxetine group were administered fluoxetine hydrochloride daily at a dose of 4 mg/kg via oral gavage. Fluoxetine hydrochloride capsules were purchased from Suzhou Zhonghua Pharmaceutical Industry Co. Ltd. (Jiangsu, China). Mice in the control and model groups received normal saline of same volume via gavage. All treatments were performed once daily for 21 consecutive days. At the end of the experimental period, mice underwent behavioral tests, followed by a 12‐h fasting period. The mice were then euthanized, and hippocampal tissue samples were collected.

### 2.5 Behavioral Tests

#### 2.5.1 Sucrose Preference Test (SPT)

During the first 24 h, mice were trained with two bottles of 1% sucrose solution, then one bottle was replaced with pure water for the next 24 h. After adaptation, mice were deprived of water for 24 h. During the SPT, each mouse was provided with one bottle of 1% sucrose solution and one bottle with pure water for 1 h. The bottles were weighed before and after the test, and the consumption of water and sucrose solution was recorded. Sucrose preference was calculated by the following formula: sucrose preference (%) = sucrose consumption/(water consumption + sucrose consumption) × 100%.

#### 2.5.2 Open Field Test (OFT)

Each mouse was placed in the center of an open‐field arena (50 × 50 × 40 cm) and allowed to explore freely for 5 min. The total distance traveled (cm) and time spent in the center of the arena (s) were recorded. The arena was wiped with 75% ethanol between trials.

#### 2.5.3 Tail Suspension Test (TST)

Mice were suspended individually by the tail using tape attached to a suspension apparatus, with the tape positioned approximately 1 cm from the tip of the tail. The test lasted for 6 min, and the immobility time during the final 4 min was recorded and analyzed.

#### 2.5.4 Elisa

The levels of hippocampus glutamate were determined with ELISA following the kit's instructions (EHJ‐96869 m, Huijia Biotechnology, China) using a microplate reader.

#### 2.5.5 Nissl Staining

Mouse brains were fixed in 4% paraformaldehyde and embedded in paraffin. After deparaffinization and rehydration, the sections were placed in a toluidine blue staining solution (G1436, Solarbio, China). Following washing, the sections were decolorized with 95% ethanol until the staining became distinct, and the nissl bodies were observed under a light microscope (Olympus, USA).

#### 2.5.6 Immunohistochemistry and Immunofluorescence

Brain sections were dewaxed, rehydrated, and subjected to antigen retrieval by heating in EDTA buffer (pH 9.0) using a pressure cooker, followed by natural cooling to room temperature. For immunohistochemistry, endogenous peroxidase activity was blocked by incubating the sections in 3% H_2_O_2_ for 30 min. The sections were then incubated with a primary antibody against GLT‐1 (HA722831, HUABIO, China) overnight at 4°C. After washing with TBST, the sections were incubated with an appropriate secondary antibody for 45 min at room temperature, and chromogenic detection was performed using a 3,3′‐diaminobenzidine (DAB) substrate kit (DAB4033, maxim Biotechnology, China). Images were captured under a light microscope (Olympus, USA).

For immunofluorescence staining, after antigen retrieval, brain sections were blocked with 10% goat serum for 30 min and then incubated with antibody against GFAP (60190‐1‐Ig, Proteintech, China) overnight at 4°C. After washing, the sections were incubated with a fluorescently labeled secondary antibody for 45 min at room temperature in the dark. Nuclei were counterstained with DAPI for 5 min. Finally, images were viewed using a fluorescence microscope (Olympus, USA).

#### 2.5.7 Western Blot

Total protein was extracted from hippocampus, using the total protein extraction kit following the manufacturer's instructions. Protein samples were separated by SDS‐PAGE and transferred to a PVDF membrane. Then the membranes were blocked with 5% non‐fat milk for 2 h at room temperature and subsequently incubated overnight at 4°C with primary antibodies against RAGE (1:2000, 16346‐1‐AP, Proteintech, China), NF‐κB p65 (1:1000, #8242, Cell Signaling Technology, USA), p‐NF‐κB p65 (1:1000, #3033, Cell Signaling Technology, USA). Following this was incubation with secondary antibodies for 1 h at room temperature. Protein bands were visible via enhanced chemiluminescence (ECL) detection reagent and a chemiluminescence imaging system, and quantified using ImageJ. The relative protein expression levels were quantified and normalized to β‐actin.

#### 2.5.8 Statistical Analysis

The experimental data are presented as the mean ± standard deviation (SD). Statistical analyses were performed using SPSS software (version 25.0). Normality was assessed using the Shapiro–Wilk test and homogeneity of variance using Levene's test. For comparisons among multiple groups, one‐way analysis of variance (ANOVA) was performed, followed by Tukey's multiple comparisons test for post hoc analysis when significant differences were identified. A *p*‐value < 0.05 was considered statistically significant.

## 3 Results

### 3.1 UPLC‐MS/MS Analysis of CHSGS Extracts

**FIGURE 1:**
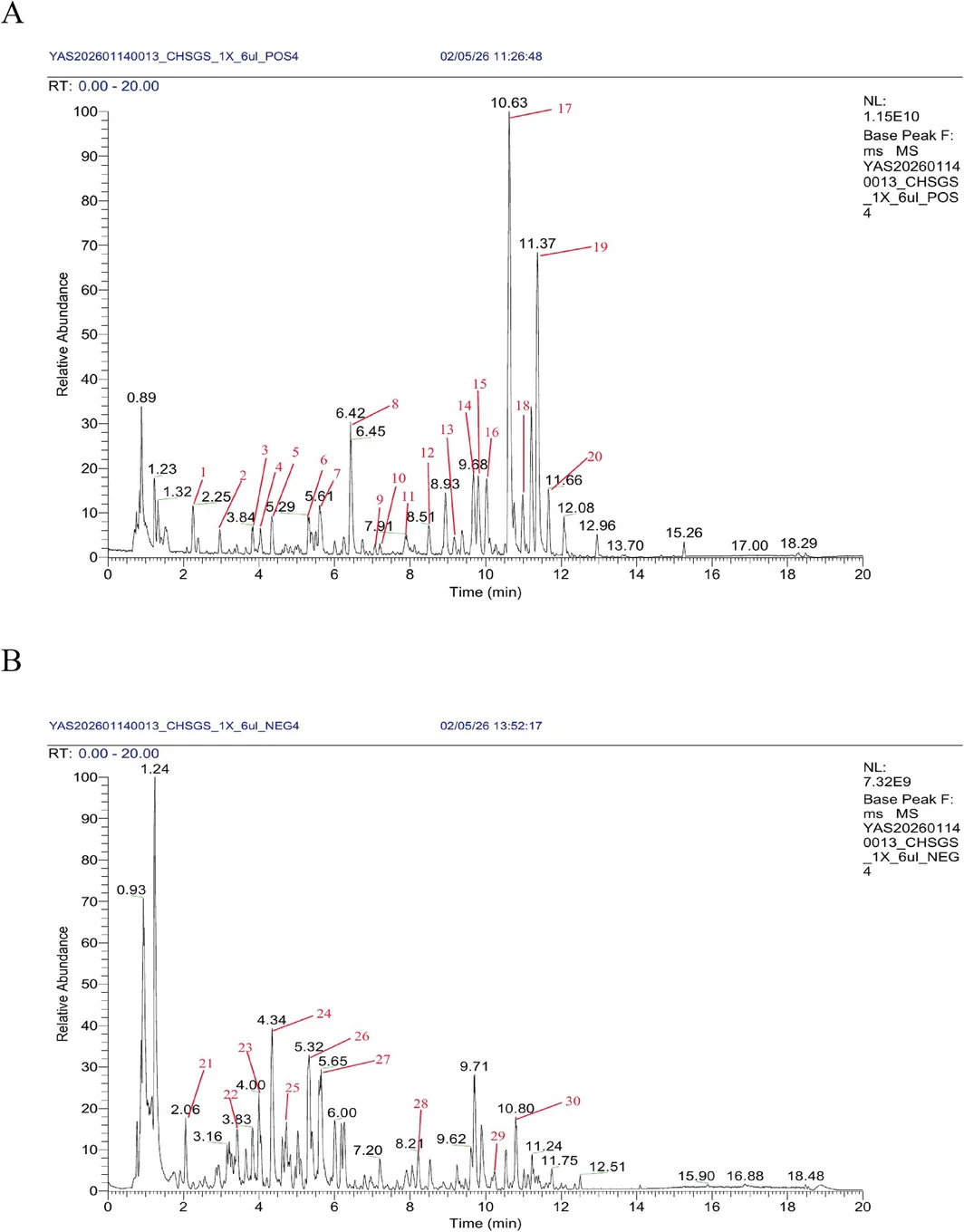
Compounds identified in CHSGS by UPLC–MS/MS analysis. (A) Base peak chromatogram of positive (+) ion modes. (B) Base peak chromatogram of negative (−) ion modes.

UPLC‐MS/MS analysis of CHSGS extracts generated a total ion chromatogram (TIC)(Figure 1). Compound identification was completed by matching accurate mass, isotopic distributions, and MS/MS spectra to reference data from a TCM database provided by Shanghai Applied Protein Technology CO. Ltd., as well as public databases including GNPS, ReSpect, and MassBank. The identified compounds covered multiple chemical classes, with flavonoids representing the predominant group, followed by coumarins, triterpenes, monoterpenes, phenolic acids, and peptides. Major identified constituents included *nobiletin*, *hesperetin*, *naringenin*, *glycyrrhizinate*, *paeoniflorin*, and *albiflorin*, among others. The identification results of 30 representative chemical constituents are summarized in Table 1.

**TABLE 1:** The identification of 30 representative chemical constituents in CHSGS by UPLC–MS/MS.

NO	RT/min	Precursor ion (m/z)	Adduct	Formula	Mass error (ppm)	Compound name
1	2.27	166.0865	[M + H‐C4H6N2O2]+	C13H17N3O4	0.6	Gly‐Gly‐Phe
2	2.97	205.0972	[M + H]+	C11H12N2O2	0.6	L‐Tryptophan
3	3.84	595.1666	[M + H]+	C27H30O15	2.4	Vicenin‐2
4	4.05	481.1709	[M + H]+	C23H28O11	1.4	Albiflorin
5	4.35	179.0704	[M + H]+	C10H10O3	0.5	Mellein
6	5.32	273.0759	[M + H‐C15H16O7]+	C30H28O12	0.3	3″‐p‐Coumaroylprunin
7	5.62	303.0865	[M + H]+	C16H14O6	1.3	Hesperetin
8	6.44	261.1123	[M + H]+	C15H16O4	2.2	Isomeranzin
9	7.08	247.0968	[M + H]+	C14H14O4	1.1	Dihydrooroselol
10	7.21	287.0916	[M + H]+	C16H14O5	1.1	Ponciretin
11	7.9	567.3507	[M + H]+	C27H46N6O7	1	Citrusin V
12	8.51	243.1018	[M‐H2O + H]+	C15H16O4	1	Auraptenol
13	9.18	217.0498	[M + H]+	C12H8O4	2.3	Majudin
14	9.71	471.2019	[M + H]+	C26H30O8	0.8	Evodin
15	9.82	373.1284	[M + H]+	C20H20O7	1.1	Sinensetin
16	10.03	343.1177	[M + H]+	C19H18O6	1.6	6‐Demethoxytangeretin
17	10.64	403.139	[M + H]+	C21H22O8	1	Nobiletin
18	11	433.1497	[M + H]+	C22H24O9	1.5	Hibiscetin heptamethyl ether
19	11.38	373.1283	[M + H]+	C20H20O7	1	Isosinensetin
20	11.67	389.1233	[M + H]+	C20H20O8	2.9	5‐O‐Demethylnobiletin
21	2.06	169.0137	[M‐H]—	C7H6O5	2.8	Gallic acid
22	3.43	165.0552	[M‐H]—	C9H10O3	2.9	(+)‐2‐Phenyllactic acid
23	4.01	167.0345	[M‐H]—	C8H8O4	2.9	3,4‐Dihydroxyphenylacetic acid
24	4.35	525.1621	[M + FA‐H]—	C23H28O11	1.3	Paeoniflorin
25	4.73	595.1679	[M‐H]—	C27H32O15	1.3	Eriocitrin
26	5.32	579.1729	[M‐H]—	C27H32O14	1.4	5‐Hydroxy‐2‐(4‐hydroxyphenyl)‐4‐oxo‐3,4‐dihydro‐2H‐chromen‐7‐yl 2‐O‐(6‐deoxy‐.alpha.‐L‐mannopyranosyl) hexopyranoside
27	5.63	609.1836	[M‐H]—	C28H34O15	1.5	Hesperetin‐7‐o‐neohesperidoside
28	8.23	271.0617	[M‐H]—	C15H12O5	2.1	Naringenin
29	10.25	837.3928	[M‐H]—	C42H62O17	1.6	Licoricesaponin g2
30	10.81	821.398	[M‐H]—	C42H62O16	1.9	Glycyrrhizinate

### 3.2 Network Pharmacology Analysis of the Antidepressant‐Like Effects of CHSGS

**FIGURE 2:**
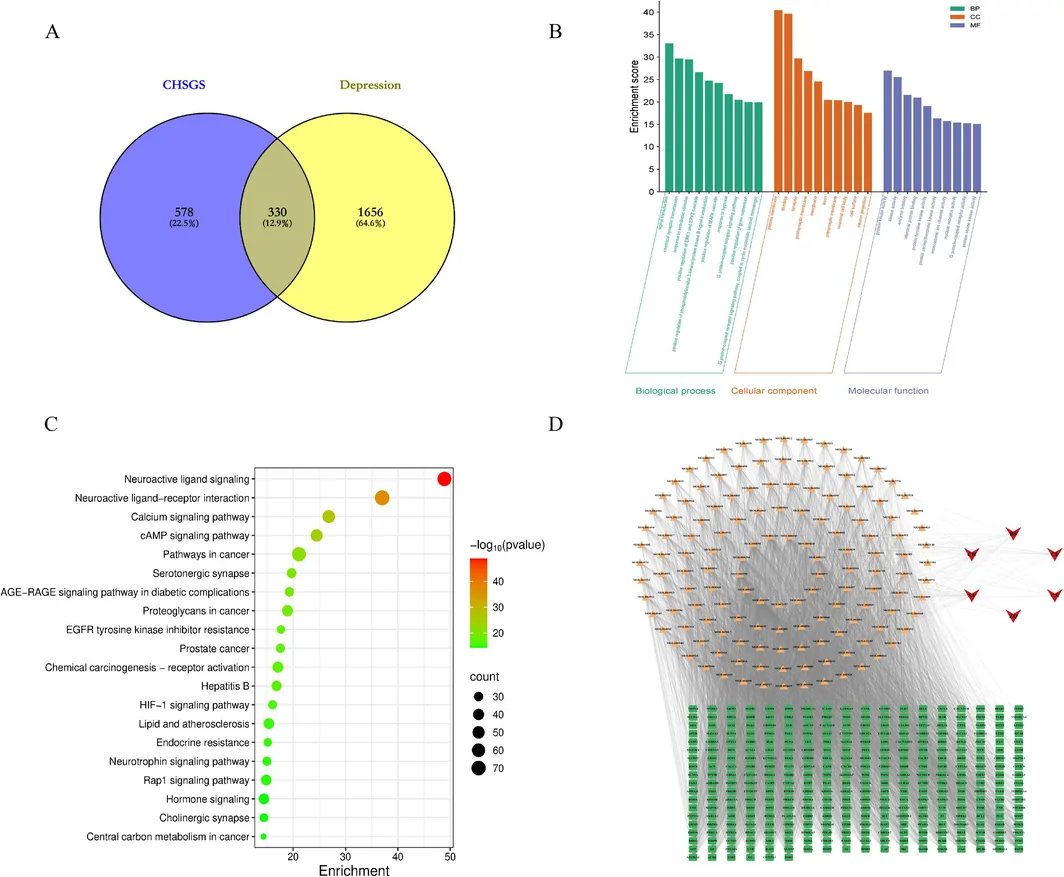
Network pharmacology analysis of the antidepressant‐like effects of CHSGS. (A) Venn diagram. (B) GO analysis of the top 10 results. (C) KEGG Pathway analysis of the top 20 results. (D) The herb–compound–target network.

To explore the potential mechanisms underlying the antidepressant‐like effects of CHSGS, an intersection analysis between CHSGS‐related targets and depression‐associated targets was performed. A total of 330 overlapping targets were identified (Figure 2A), which were considered potential molecular targets underlying the antidepressant‐like effects of CHSGS. GO enrichment analysis revealed that the intersecting targets are primarily involved in biological processes such as signal transduction, chemical synaptic transmission, responses to exogenous stimuli, and the upregulation of the ERK1/ERK2 and PI3K/Akt signaling pathways. In terms of cellular components, the targets were predominantly associated with the plasma membrane, dendrites, synapses, and postsynaptic membranes. Molecular function analysis revealed enrichment in protein kinase activity, kinase activity, enzyme binding, and protein binding (Figure 2B). KEGG pathway enrichment analysis revealed that the intersection targets were significantly enriched in pathways such as circadian rhythm, cancer‐related pathways, serotonin synthesis, cAMP, AGE–RAGE, HIF‐1, and Rap1 (Figure 2C). Among these significantly enriched pathways, the AGE–RAGE signaling pathway, which has been reported to be associated with glutamate homeostasis, was prioritized for subsequent investigation of the antidepressant‐like effects of CHSGS.

Furthermore, the integrated herb–compound–target network was constructed to show the characteristics of CHSGS in the treatment of depression (Figure 2D).

### 3.3 Molecular Docking Analysis

**FIGURE 3:**
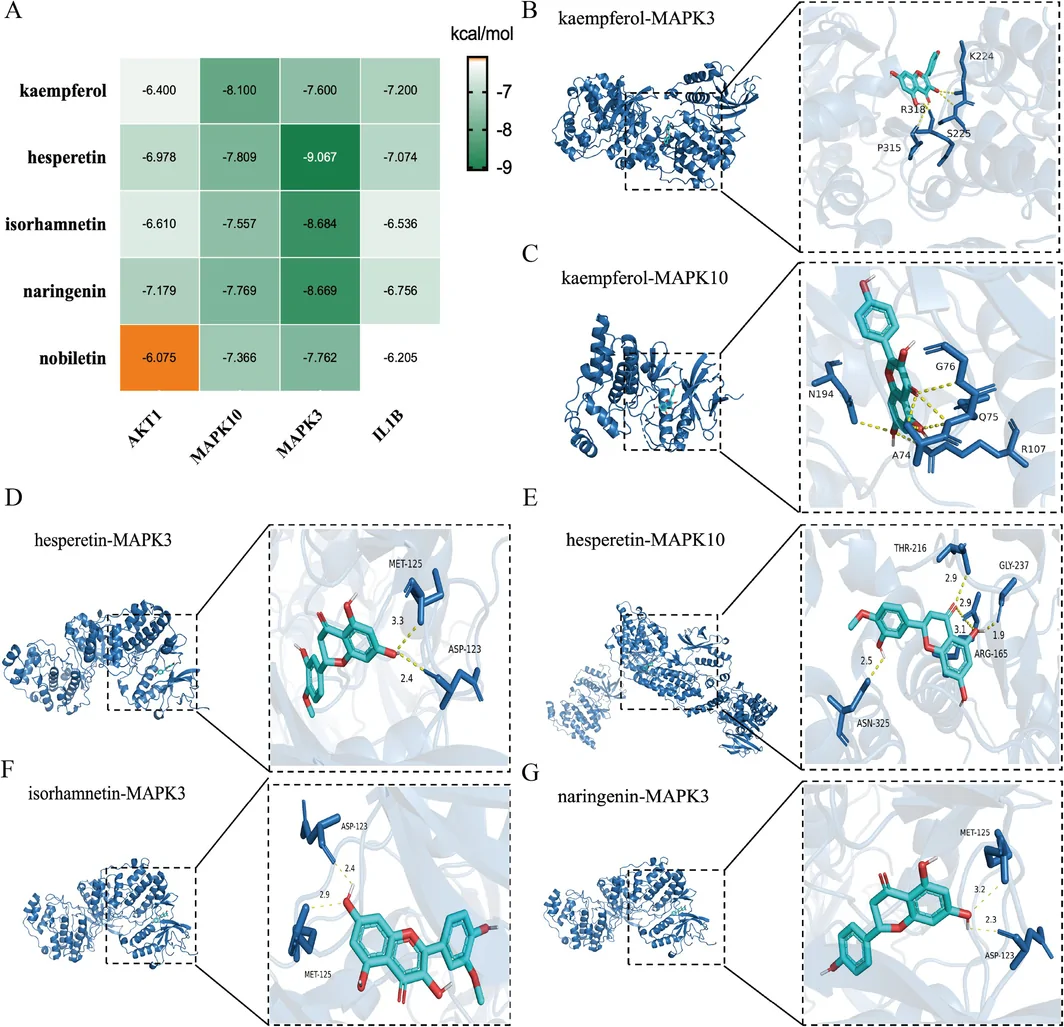
The docking scores and visualization of key targets with their corresponding active compounds. (A) The docking scores. (B) *kaempferol*‐MAPK3 complex. (C) *kaempferol*‐MAPK10 complex. (D) *hesperetin*‐MAPK3 complex. (E) *hesperetin*‐MAPK10 complex. (F) *isorhamnetin*‐MAPK3 complex. (G) *naringenin*‐MAPK3 complex.

To further explore the interactions between the major active compounds of CHSGS and the predicted therapeutic targets, five compounds detected in the CHSGS extract by UPLC–MS/MS, including *kaempferol, hesperetin, isorhamnetin, naringenin*, and *nobiletin*, were selected for molecular docking with four proteins associated with the AGE–RAGE signaling pathway, namely AKT1, MAPK10, MAPK3, and IL1B. The docking scores are summarized in Figure 3A. In general, lower docking scores indicate more favorable predicted ligand–protein interactions. The selected compounds exhibited favorable docking scores with the target proteins, with several compound–MAPK3 and compound–MAPK10 combinations showing particularly favorable docking scores (Figure 3B–G). These findings suggest that the identified compounds may interact with key proteins involved in AGE–RAGE signaling, providing further support for the potential involvement of this pathway in the pharmacological effects of CHSGS.

### 3.4 CHSGS Treatment Ameliorated CRS‐Induced Behavioral Abnormalities in Mice

**FIGURE 4:**
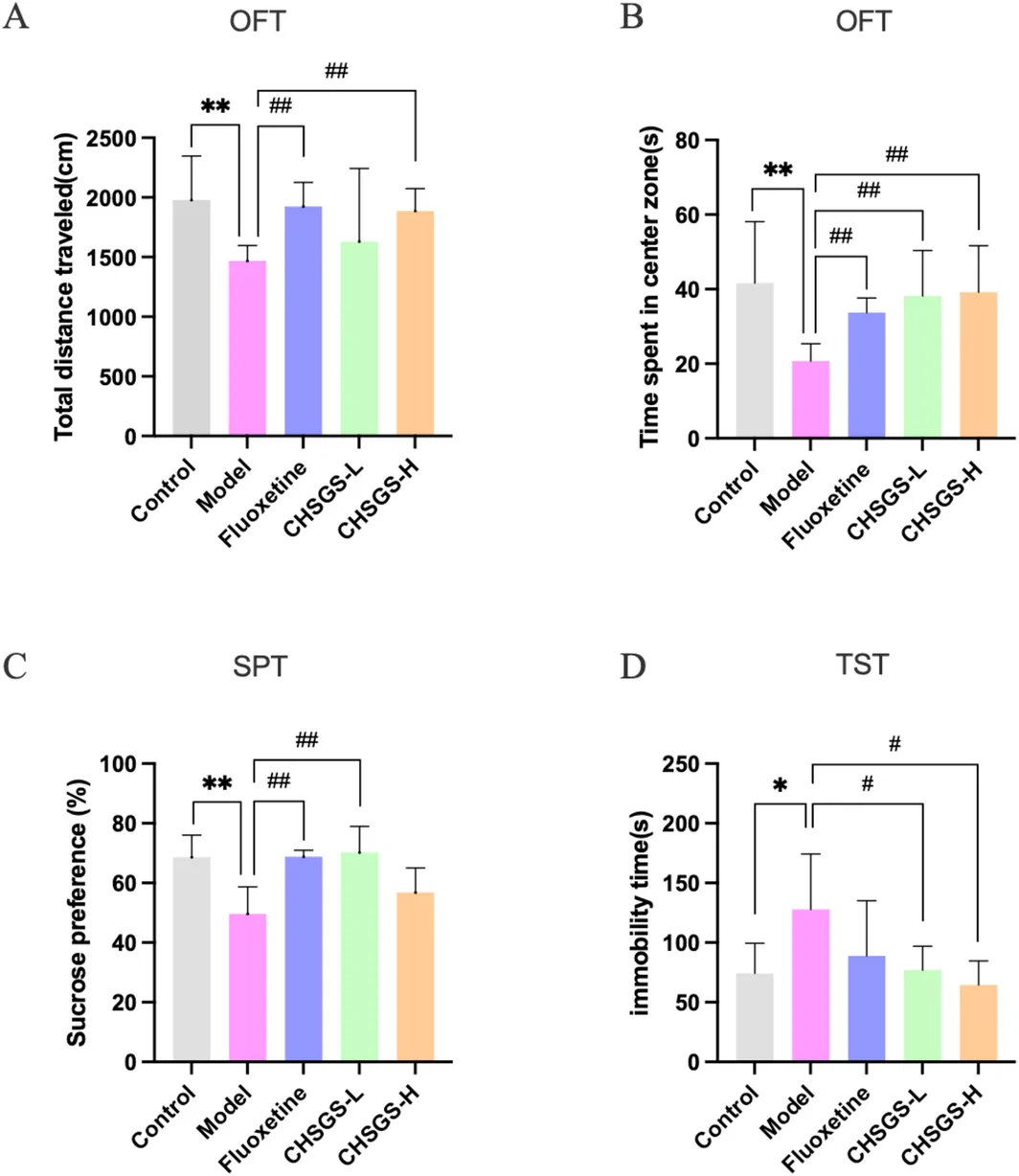
CHSGS treatment ameliorated CRS‐induced behavioral abnormalities in mice. (A) Total distance traveled in OFT. (B) Time spent in the center zone in OFT. (C) Sucrose preference in SPT. (D) Immobility time in TST. Values were expressed as the mean ± standard deviation (X¯ ± SD) (*n* = 6). *p < 0.05, **p < 0.01 compared with the control group. ^#^ *p* < 0.05, ^##^ *p* < 0.01 compared with the model group.

To evaluate the effects of CHSGS on CRS‐induced behavioral abnormalities, mice were subjected to behavioral tests after CRS exposure and treatment (Figure 4). Compared with the control group, CRS exposure induced significant behavioral alterations, including reduced sucrose preference, decreased total distance traveled and time spent in the central zone in the open‐field test, and increased immobility time in the tail suspension test (*p* < 0.01 or *p* < 0.05). CHSGS treatment partially ameliorated these behavioral alterations. High‐dose CHSGS significantly increased the time spent in the central zone and total distance traveled in the open‐field test (*p* < 0.01), whereas low‐dose CHSGS significantly increased only the time spent in the central zone (*p* < 0.01). Low‐dose CHSGS also significantly increased sucrose preference (*p* < 0.01), and both CHSGS doses reduced immobility time in the tail suspension test (*p* < 0.05). Fluoxetine significantly improved sucrose preference and open‐field performance (*p* < 0.01), whereas no significant difference was observed in immobility time.

### 3.5 CHSGS Reduced Hippocampal Glutamate Accumulation and Ameliorated CRS‐Associated Neuronal Morphological Alterations

**FIGURE 5:**
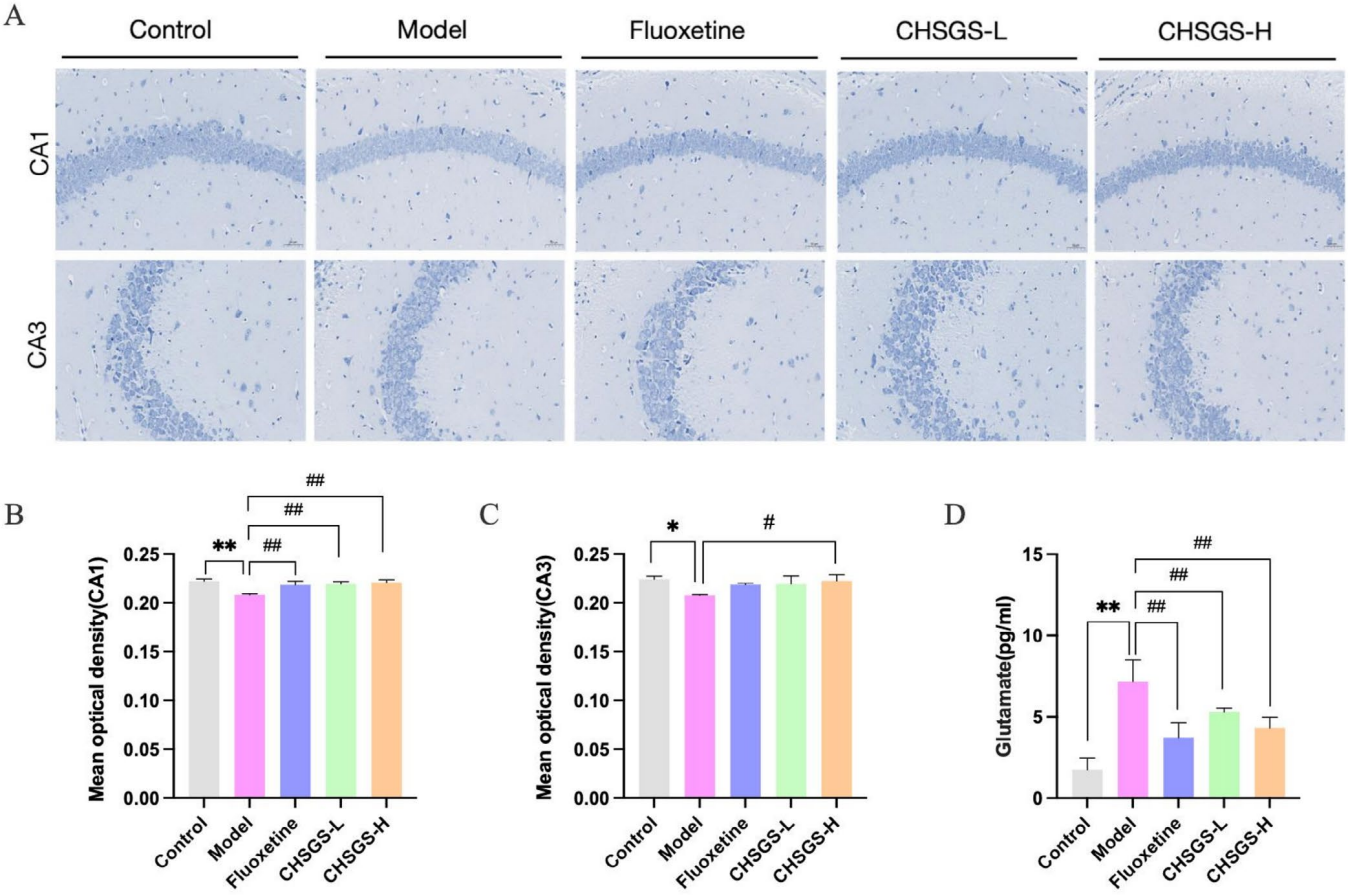
CHSGS reduced hippocampal glutamate accumulation and ameliorated neuronal morphological alterations in CRS mice. (A) Nissl bodies in hippocampus CA1 and CA3 (scale bar = 50 μm, *n* = 3). (B) Quantification of MOD in the CA1 region. (C) Quantification of MOD in the CA3 region. (D) Levels of glutamate in hippocampus (*n* = 6). *p < 0.05, **p < 0.01 compared with the control group. ^#^ *p* < 0.05, ^##^ *p* < 0.01 compared with the model group. Values were expressed as the mean ± standard deviation (X¯ ± SD).

Neuronal morphology was assessed using Nissl staining (Figure 5). In the control group, the neuronal structure was intact, with abundant, deeply stained Nissl bodies in the cytoplasm. In contrast, CRS mice exhibited neuronal morphological alterations characterized by reduced Nissl body density and staining intensity, accompanied by decreased mean optical density (MOD) values in the hippocampal CA1 and CA3 regions compared with the control group (*p* < 0.01 or *p* < 0.05). In addition, hippocampal glutamate levels were significantly increased in CRS‐exposed mice (*p* < 0.01). Following CHSGS or Fluoxetine treatment, neuronal morphology was improved, with more abundant and intensely stained Nissl bodies compared with the model group. Both doses of CHSGS significantly increased the MOD in the CA1 region (*p* < 0.01), whereas only high‐dose CHSGS significantly increased the MOD in the CA3 region (*p* < 0.05). Fluoxetine also significantly increased the CA1 MOD (*p* < 0.01), but had no significant effect on the CA3 region. Consistent with these findings, both doses of CHSGS and fluoxetine significantly reduced hippocampal glutamate levels (*p* < 0.01), indicating that CHSGS reduced hippocampal glutamate accumulation and was associated with an improvement in CRS‐related neuronal morphological alterations.

### 3.6 CHSGS Modulated Hippocampal GFAP and GLT‐1 Expression in CRS Mice

**FIGURE 6:**
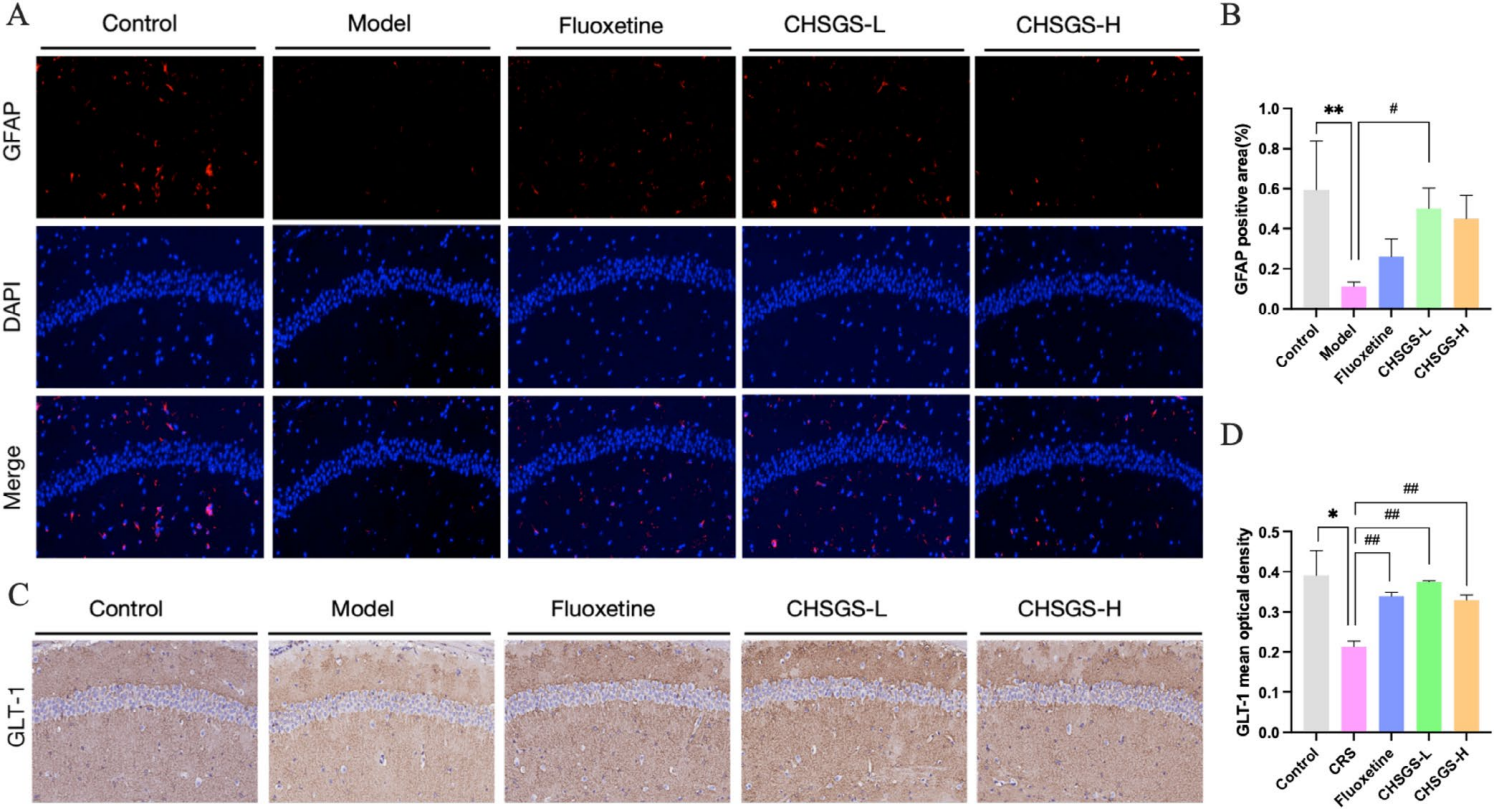
CHSGS modulated hippocampal GFAP and GLT‐1 expression in CRS mice. (A, B) Immunofluorescence staining and quantification of GFAP in CA1 (scale bar = 50 μm, *n* = 3).(C, D) Immunohistochemical staining and quantification of GLT‐1 in CA1 (scale bar = 50 μm, *n* = 3). Values were expressed as the mean ± standard deviation (X¯ ± SD). *p < 0.05, **p < 0.01 compared with the control group. #*p* < 0.05, ##*p* < 0.01 compared with the model group.

To further investigate astrocyte‐associated changes and glutamate transporter expression following CHSGS treatment, hippocampal GFAP immunofluorescence and GLT‐1 immunohistochemical staining were performed (Figure 6). Compared with the control group, CRS mice exhibited significantly reduced hippocampal GFAP and GLT‐1 expression (*p* < 0.01, *p* < 0.05). Following treatment, both doses of CHSGS and fluoxetine significantly increased GLT‐1 expression (*p* < 0.01). In addition, low‐dose CHSGS significantly increased GFAP expression (*p* < 0.05), whereas the increases observed in high‐dose CHSGS and fluoxetine groups did not reach statistical significance. These findings suggest that CHSGS modulated CRS‐associated changes in hippocampal GFAP and GLT‐1 expression.

### 3.7 CHSGS Modulated Hippocampal RAGE/NF‐κB Signaling in CRS Mice

**FIGURE 7:**
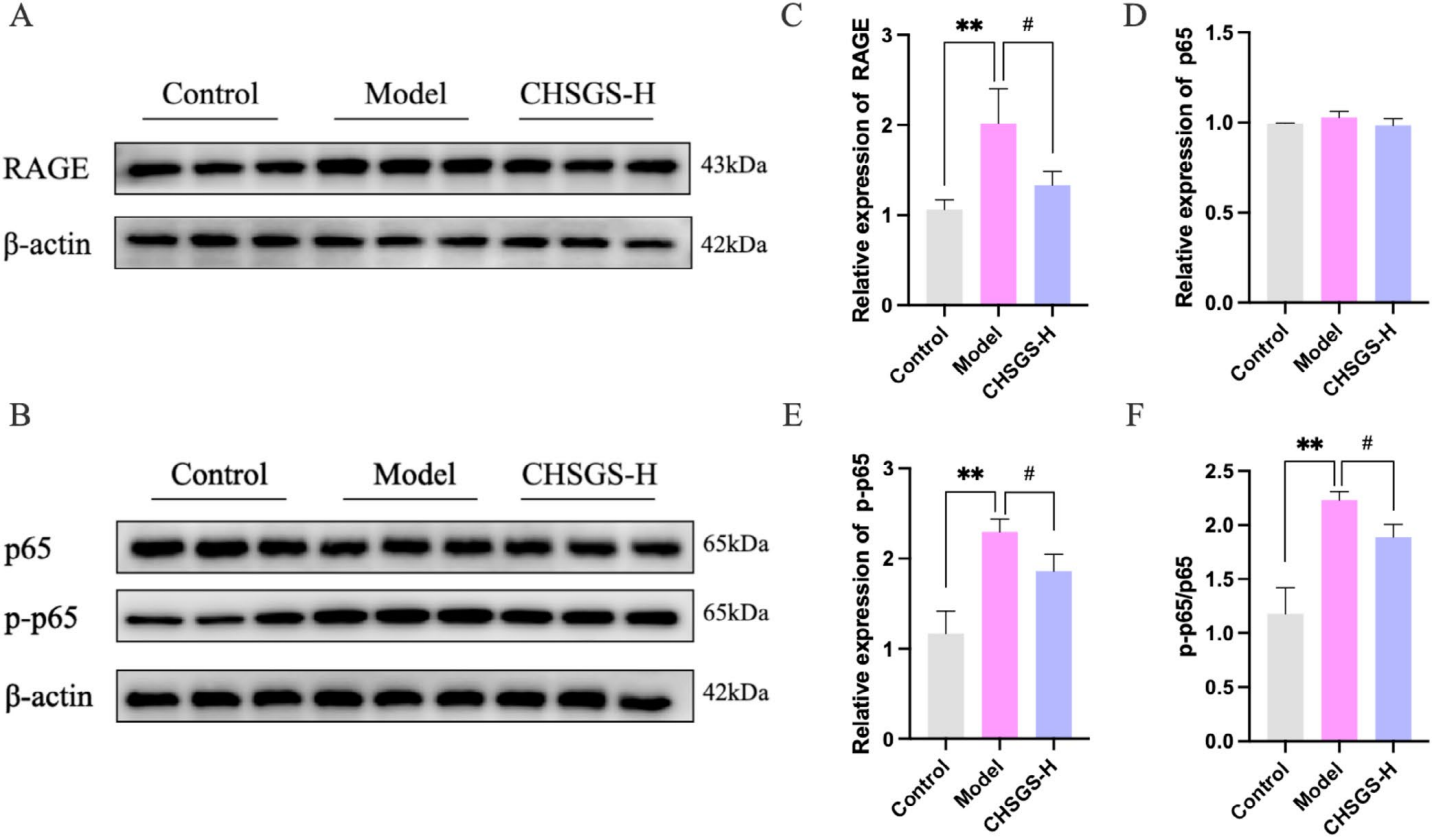
CHSGS modulated the expression of hippocampal RAGE and NF‐κB in CRS mice. (A) Expression bands of RAGE protein. (B) Expression bands of p65 and p‐p65 proteins. (C–F) Protein expression levels of RAGE, p65, and p‐p65, and the p‐p65/p65 ratio. Values were expressed as the mean ± standard deviation (X¯ ± SD) (*n* = 3). *p < 0.05, **p < 0.01 compared with the control group. #*p* < 0.05 compared with the model group.

To validate the involvement of the AGE–RAGE signaling pathway predicted by network pharmacology, the expression levels of RAGE, NF‐κB p65, phosphorylated NF‐κB p65 (p‐p65), and the p‐p65/p65 ratio were detected in the hippocampus of CRS mice (Figure 7). Compared with the control group, the expression of RAGE, p‐p65, and the p‐p65/p65 ratio were significantly increased in the hippocampus of CRS mice (*p* < 0.01), whereas total p65 expression showed no significant difference. Following CHSGS treatment, both RAGE expression, p65 phosphorylation, and the p‐p65/p65 ratio were significantly reduced compared with those in the model group (*p* < 0.05), indicating inhibition of CRS‐induced activation of the RAGE/NF‐κB signaling pathway.

## 4 Discussion

The present study suggests that CHSGS ameliorates CRS‐induced behavioral abnormalities in mice. CHSGS treatment also reduced hippocampal glutamate levels, improved neuronal morphology, increased GFAP and GLT‐1 expression, and attenuated RAGE/NF‐κB signaling activation. Collectively, these findings suggest that the antidepressant‐like effects of CHSGS may involve the restoration of hippocampal glutamate homeostasis.

Disrupted glutamate homeostasis is associated with neuronal dysfunction under chronic stress conditions (Wang et al. 2017; Duman et al. 2019; Avalos et al. 2022; Zhang et al. 2026). In the present study, CRS increased hippocampal glutamate levels and induced alterations in neuronal Nissl staining, whereas CHSGS reduced glutamate accumulation and improved neuronal morphology. These results suggest that regulation of hippocampal glutamate homeostasis may contribute to the effects of CHSGS. This interpretation is also consistent with previous reports that CHSGS can influence glutamate receptor activation and synaptic protein expression under chronic stress conditions (Lu et al. 2024).

GLT‐1 plays a central role in maintaining extracellular glutamate homeostasis by transporting glutamate into astrocytes and supporting the glutamate–glutamine cycle (Park and Choi 2025). Reduced GLT‐1 expression has been associated with extracellular glutamate accumulation and neuronal injury in stress‐related models (Codeluppi et al. 2021; Wang et al. 2024; Prevot et al. 2025; Cheng et al. 2026). In the present study, CHSGS increased hippocampal GLT‐1 expression in parallel with a reduction in glutamate levels. The GFAP results also indicated a partial improvement in CRS‐associated astrocytic alterations. These coordinated changes support an association between CHSGS treatment and improved glutamate homeostasis.

Network pharmacology identified AGE–RAGE signaling as a candidate pathway. Since NF‐κB is a key downstream mediator of RAGE activation, we further examined RAGE/NF‐κB signaling in vivo. CRS increased hippocampal RAGE expression and NF‐κB p65 phosphorylation, whereas CHSGS reduced these changes. Previous studies have associated sustained RAGE/NF‐κB activation with neuroinflammation, neuronal injury, and stress‐related behavioral abnormalities (Li et al. 2021, 2025; Yu et al. 2022; Bhattacharya et al. 2023). NF‐κB signaling has also been implicated in the transcriptional regulation of GLT‐1. The promoter region of the GLT‐1 gene contains multiple NF‐κB‐binding sites, and NF‐κB has been reported to regulate GLT‐1 transcription through response elements within its promoter. However, this regulation appears to be context dependent. Under pathological conditions, such as chronic stress, sustained NF‐κB activation may also suppress GLT‐1 expression, potentially through the recruitment of transcriptional corepressors (Sitcheran et al. 2005; Ghosh et al. 2011; Karki et al. 2013; Yang et al. 2020; Liu et al. 2022). In this study, the concurrent changes in RAGE/NF‐κB signaling, GLT‐1 expression, and hippocampal glutamate levels suggest that these processes may be associated with the effects of CHSGS on glutamate homeostasis.

UPLC–MS/MS profiling identified multiple classes of constituents in CHSGS, among which flavonoids were predominant. Flavonoids are naturally occurring polyphenolic compounds widely distributed in vegetables, fruits, grains, and tea. They have been reported to exhibit anti‐inflammatory, antioxidant, antidepressant‐like, neurotransmitter‐modulating, and neuroprotective activities (Pannu et al. 2021; Jia et al. 2023). Molecular docking analysis further suggested potential interactions between several major flavonoids, including *kaempferol, hesperetin, isorhamnetin, naringenin*, and *nobiletin*, and proteins involved in AGE–RAGE signaling. Some of these compounds have also been reported to exert neuroprotective effects by reducing excessive glutamate accumulation and modulating glutamate receptor activation (Yang et al. 2014; Luo et al. 2021; Jahan et al. 2022; Alqudah et al. 2025). Although the current evidence is primarily derived from computational analyses, our in vivo findings provide additional support for the potential multicomponent and multitarget properties of CHSGS.

However, several limitations should be acknowledged. Although our findings suggest that modulation of RAGE/NF‐κB signaling and GLT‐1 expression may be associated with the effects of CHSGS, further studies employing selective pathway inhibitors are required to clarify the causal relationships among these molecular changes, glutamate regulation, and behavioral improvement. In addition, GFAP immunofluorescence and GLT‐1 immunohistochemical staining provided evidence of astrocyte‐associated alterations; however, further functional studies are needed to clarify the role of astrocytic GLT‐1 in glutamate regulation.

## 5 Conclusion

In summary, CHSGS ameliorated CRS‐induced behavioral abnormalities in mice. These effects were accompanied by reduced hippocampal glutamate levels, improved neuronal morphology, increased GFAP and GLT‐1 expression, and suppressed activation of the RAGE/NF‐κB signaling pathway. These findings suggest that restoration of hippocampal glutamate homeostasis may contribute to the antidepressant‐like effects of CHSGS, which may be associated with the regulation of GLT‐1 expression and involvement of the RAGE/NF‐κB signaling pathway. This study provides experimental evidence supporting the regulation of glutamate homeostasis as a potential pharmacological basis for the antidepressant‐like effects of CHSGS.

## Author Contributions

**Yu Xu:** conceptualization, writing – original draft, funding acquisition. **Lian Xia:** methodology, validation, writing – original draft, visualization. **Keyun Zhou:** validation, formal analysis. **Chuanxin Liu:** validation, formal analysis. **Xiaoting Tian:** methodology, formal analysis, resources, writing – review and editing. **Zongchun Yang:** conceptualization, methodology, writing – review and editing, visualization, funding acquisition.

## Funding

This work was supported by Natural Science Foundation of Chongqing, China, CSTB2022NSCQ‐MSX1640, CSTB2022NSCQ‐MSX1562.

## Conflicts of Interest

The authors declare no conflicts of interest.

## Data Availability

The data that support the findings of this study are available from the corresponding author upon reasonable request.
